# AI Models to Assist Vancomycin Dosage Titration

**DOI:** 10.3389/fphar.2022.801928

**Published:** 2022-02-08

**Authors:** Zhiyu Wang, Chiat Ling Jasmine Ong, Zhiyan Fu

**Affiliations:** ^1^ Integrated Health Information Systems (IHIS), Singapore, Singapore; ^2^ Department of Pharmacy, Singapore General Hospital, Singapore, Singapore

**Keywords:** machine learning, artificial intelligence, vancomycin, dosage titration, dosing recommendation, therapeutic drug monitoring

## Abstract

**Background:** Effective treatment using antibiotic vancomycin requires close monitoring of serum drug levels due to its narrow therapeutic index. In the current practice, physicians use various dosing algorithms for dosage titration, but these algorithms reported low success in achieving therapeutic targets. We explored using artificial intelligent to assist vancomycin dosage titration.

**Methods:** We used a novel method to generate the label for each record and only included records with appropriate label data to generate a clean cohort with 2,282 patients and 7,912 injection records. Among them, 64% of patients were used to train two machine learning models, one for initial dose recommendation and another for subsequent dose recommendation. The model performance was evaluated using two metrics: PAR, a pharmacology meaningful metric defined by us, and Mean Absolute Error (MAE), a commonly used regression metric.

**Results:** In our 3-year data, only a small portion (34.1%) of current injection doses could reach the desired vancomycin trough level (14–20 *mcg/ml*). Both PAR and MAE of our machine learning models were better than the classical pharmacokinetic models. Our model also showed better performance than the other previously developed machine learning models in our test data.

**Conclusion:** We developed machine learning models to recommend vancomycin dosage. Our results show that the new AI-assisted dosage titration approach has the potential to improve the traditional approaches. This is especially useful to guide decision making for inexperienced doctors in making consistent and safe dosing recommendations for high-risk medications like vancomycin.

## Introduction

Vancomycin is a glycopeptide antibiotic commonly used in the treatment of Gram-positive infections, especially methicillin-resistant *Staphylococcus aureus* (MRSA). Infections with MRSA can lead to serious complications including endocarditis, pneumonia, and skin and soft tissue infections (Hidayat et al., 2006). Currently, MRSA is endemic in hospitals around the world and accounts for significant morbidity and mortality, as well as healthcare-associated costs (Lodise Jr and McKinnon, 2007). Vancomycin therapy requires close monitoring of serum drug levels in view of its narrow therapeutic index. High serum drug levels increase the risk for nephrotoxicity, while low serum drug levels lead to reduced efficacy. The current practice recommends regular monitoring of serum trough levels at a steady state, aiming at a narrow range of 15–20 mg/L for the treatment of MRSA infections. In the latest guidelines on therapeutic drug monitoring of vancomycin, an AUC (area under curve)-guided dosing and monitoring approach was recommended using an individualized target of the AUC/MIC ratio of 400–600 (Rybak et al., 2020). However the AUC-guided approach may be relatively impractical in clinical practice (NB, 2020) and most institutions in Singapore are still using trough vancomycin levels.

In the current practice, most physicians and pharmacists use standard weight-based dosing recommendations (Lexi-drugs, 2015; Micromedex-Solution, 2021) to determine suitable initial dose for patients requiring vancomycin. These dosing recommendations usually take into consideration patient’s weight and renal function to account for differences in drug clearance. After serum trough levels are available, dosage adjustments are often arbitrary. Some institutions developed their own dosing nomogram in order to standardize dosing adjustments according to the individual patients’ serum trough concentration (Kullar et al., 2011; Thalakada et al., 2012; Kosmisky et al., 2015; Bowers et al., 2018). These dosing algorithms typically reported low to moderate success in achieving their therapeutic target of 15–20 mg/L in their validation cohorts, ranging from 35.4 to 56%. Another method was to use population pharmacokinetic models to predict vancomycin serum trough levels given a particular dosing regimen is used. The population pharmacokinetics of vancomycin has been extensively researched in different patient populations including pediatric and CKD patients. The accuracy of various models varied widely ranging from 40 to 90% (Monteiro et al., 2018; Kim et al., 2019; Lin et al., 2021). However, clinical application of these population pharmacokinetic models is limited as these models perform best in relatively homogenous patient populations.

Machine learning or artificial intelligent (AI) is a new and emerging approach for dosage titration. Owing to the large heterogeneity of dosage requirements between individual patients, machine learning methods are able to process large amounts of patient data and translate into useful clinical recommendations (Goh et al., 2020). Although a couple of studies published promising results very recently (Imai et al., 2020; Huang et al., 2021), AI-assisted vancomycin dosage titration is not widely used as a regular practice in hospitals. Our models contribute to this area in three aspects: First, the models give direct suggestions of appropriate daily dose not only to initial injection but also to subsequent injections. Second, the models are applicable to all patients, with no restriction on patients’ hemodialysis, estimated glomerular filtration rate (eGFR), and weight. Third, we developed an innovative method to derive the label data, that is, the target daily dose. The main focus of this study is about how we develop this machine learning algorithms to recommend vancomycin dosage in patients admitted to a tertiary general hospital.

## Methods

This study was exempted from institutional ethics review as no patient identifiable information was used in the data analysis.

### Data

This study used data in SingHealth-IHiS Electronic Health Intelligence System of Singapore (eHints). Data has been cleaned, consolidated, and standardized in eHints, which make it easier to access and analyze for hospital management and clinical researchers.^1^

Inpatients, who received at least one vancomycin injection during the period from January 1, 2017 to December 31, 2019, were selected. Patient’s demographics, lab test results, diagnosis, medication, and clinical treatment have been collected.

### Target Daily Dose and Acceptable Daily Dose Range of Vancomycin Injection

**FIGURE 1:**
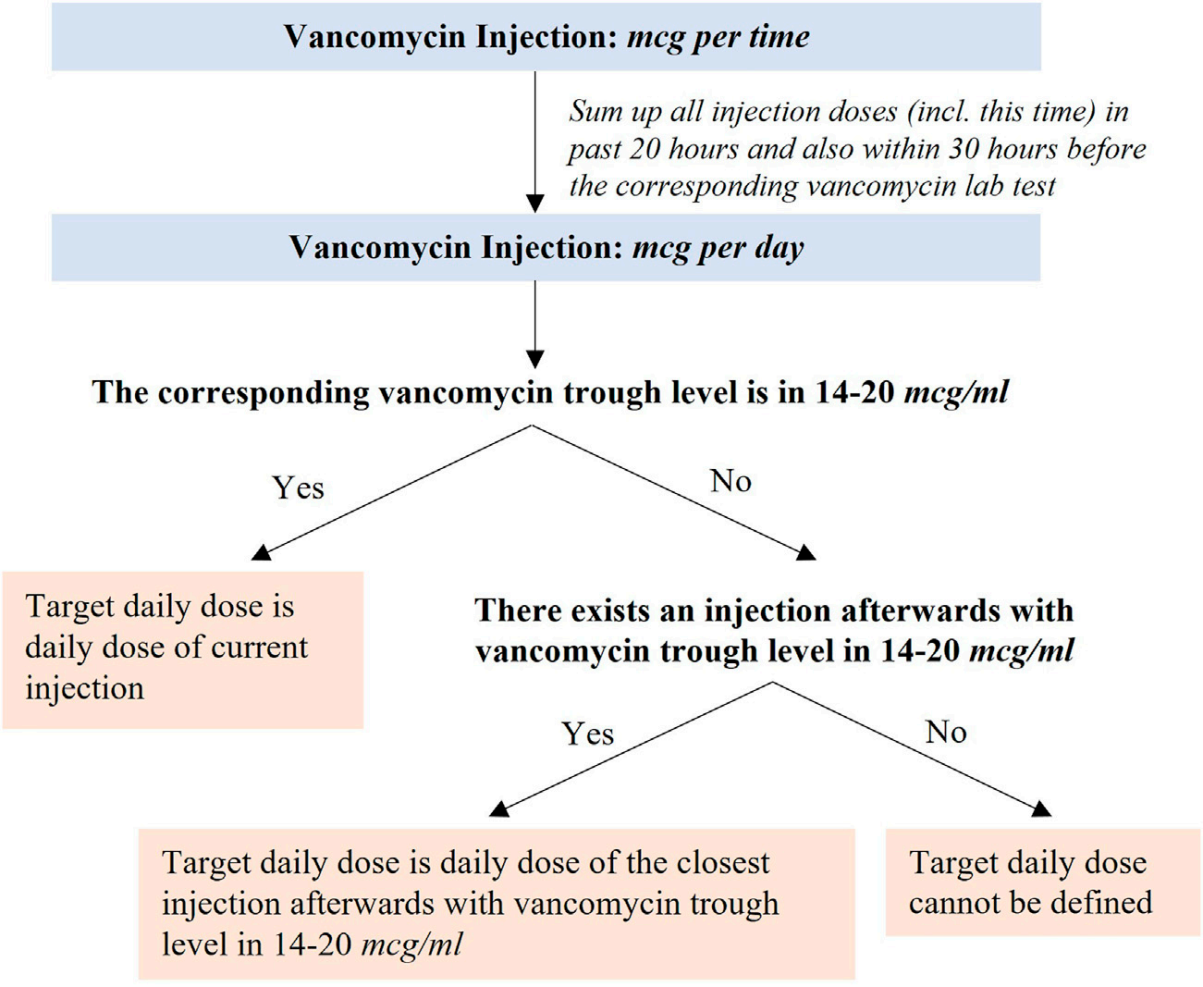
Definition of the target daily dose of injection.

A derived label for training the machine learning model, named the target daily dose, was generated using a novel method, as illustrated in Figure 1. First, we converted the unit from dose per time (mg/time) to dose per day (mg/day) for each injection by summing up all doses injected in the past 20 h (including this time) but excluding those more than 30 h before the corresponding vancomycin lab test (see Supplementary Appendix 1 for the definition on the corresponding vancomycin lab test). Next, the target daily dose was set respectively in two scenarios: 1) if the vancomycin trough level was 14–20 *mcg/ml*, then the target daily dose was the daily dose of current injection; 2) otherwise, the target daily dose was the daily dose of its closest injection afterward, if any, that drives the vancomycin trough level to 14–20 *mcg/ml*. We set our target levels to be between 14 and 20 *mcg/ml* to reflect clinical practice in our institution.

Given that the therapeutic range of the vancomycin trough level allows for some variation of vancomycin injections, the acceptable daily dose range of injections was introduced here. Any injection dose in that range was most likely to cause the desirable therapeutic effect. The calculation of the acceptable daily dose range is defined as follows:

Lower Bound of Acceptable Range=min(Target Daily Dose ×85%, Target Daily Dose−250 mg),

Upper Bound of Acceptable Range=max(Target Daily Dose ×115%, Target Daily Dose+250 mg).

### Cohort

**FIGURE 2:**
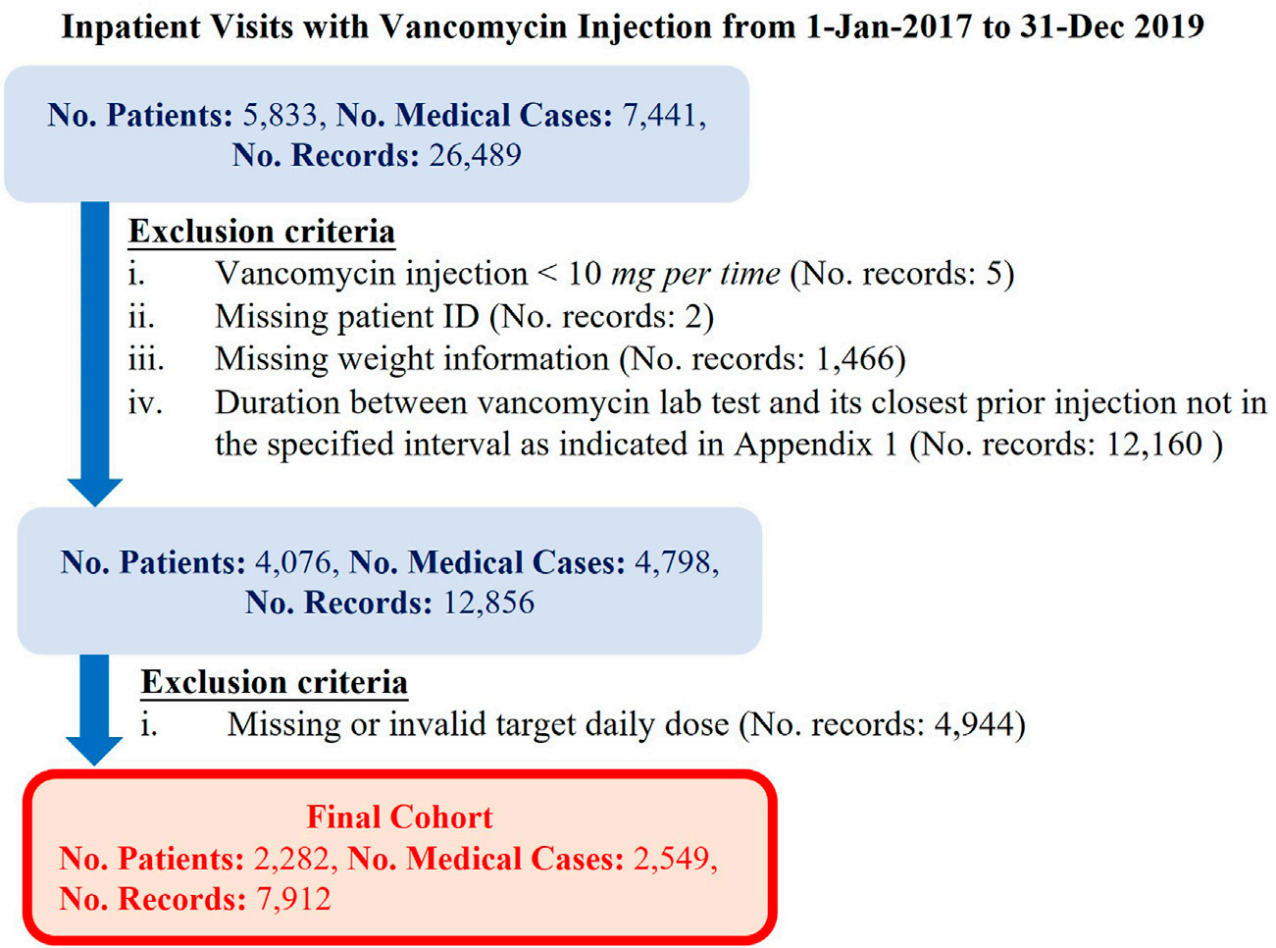
Definition of the cohort in the study.

Our cohort were the patients who were inpatient and received vancomycin injections from January 1, 2017 to December 31, 2019 in eHints. We also excluded the data satisfying any of the criteria elaborated in Figure 2: 1) vancomycin injection per time < 10 *mg*; 2) missing patient ID; 3) missing weight information; 4) the duration between vancomycin lab test and its closest prior injection was outside the range of 5–28 h; 5) the duration between vancomycin lab test and its closest injection was not in the specified interval (see the Supplementary Appendix); and 6) missing or invalid target daily dose. Invalid target daily dose had two scenarios, that is, the target daily dose is greater than the current daily dose with vancomycin trough levels greater than 20 *mcg/ml*, and the target daily dose is smaller than the current daily dose with vancomycin trough levels smaller than 14 *mcg/ml*.

### Model Development

We developed separate daily dose recommenders for the initial dose and the subsequent dose of vancomycin. The initial dose referred to those without prior vancomycin lab tests but may or may not have prior vancomycin injection. We built LightGBM models in python and fine-tune hyper-parameters in two steps. In the first step, Bayesian optimization was used. This method keeps track of previous evaluation results and applies a Tree-structured Parzen Estimator from standard Sequential Model-based Optimization to build a probabilistic model that maps the hyper-parameters to the objective function (Bergstra et al., 2011; Bergstra et al., 2013). It is efficient to select the next hyper-parameters by making fewer calls to the objective function. Bayesian optimization was implemented in the open-source python package hyperopt (http://hyperopt.github.io/hyperopt/). In the second step, we used grid search on several combinations of hyper-parameter values suggested in the first step to further improve model performance. The final values of the hyper-parameters which generated the best performance on the validation data were selected. These hyper-parameters included the number of estimators n_estimator, the max depth of each decision tree m_depth, L1 regularization reg_alpha, L2 regularization reg_lambda, learning rate learning_rate, the fraction of features (randomly selected) to train each tree colsample_bytree, and the fraction of features (randomly selected) to train each tree colsample_bylevel. The hyper-parameters control the overfitting and the learning process of models.

The study data was randomly split into three sets by patient ID: training, validation, and test. As shown in Table 1, 64% of patients were in the training set, 16% in the validation set, and 20% in the test set. There was no overlap of patients among these three sets. Among 7,912 records, 25% of them were initial doses before which there were no previous vancomycin lab tests, and 75% were subsequent doses which had previous injections available with corresponding vancomycin lab tests. The training set was to train the models, the validation set was to *fine-tune* the hyper-parameters, and the test set was to evaluate the model performance.

**TABLE 1:** Distribution of patients and records in different data sets.

Data	No. of patients	No. of records		
		Total	Initial dose	Subsequent dose
Training	1,460	5,105	1,278	3,827
Validation	366	1,164	312	852
Test	456	1,643	389	1,254
Total	2,282	7,912	1,979	5,933

LightGBM can provide the importance score of each feature it used. To interpret individual feature impact on the final model, SHAP (Lundberg and Lee, 2017) was also used to visualize the feature impact results.

### Model Evaluation

Mean absolute error (MAE) and percentage of dose in the acceptable range (PAR) were used to evaluate model performance in this study. MAE measured the deviation of the suggested dose by the model from the target daily dose. PAR took into account the scenario that the suggested dose by the model, although different from the target daily dose, still drove vancomycin trough levels into the desired range. It focused on whether injections caused a therapeutic effect instead of hitting the target daily dose precisely. The calculation of MAE and PAR is as follows:

$$\text{MAE}=\frac{1}{N}\overset{N}{\underset{i=1}{\sum }}\left|y_{i}-\hat{y_{i}}\right|,\;$$

$$\text{PAR}=\frac{1}{N}\overset{N}{\underset{i=1}{\sum }}f\left(r_{i},\;\hat{y_{i}}\right),$$

where $f\left(r_{i},\;\hat{y_{i}}\right)=1$ if $\hat{y_{i}}\in r_{i}$ else 0; $r_{i}$ is the acceptable daily dose range of vancomycin injection, $y_{i}$ is the target daily dose, $\hat{y_{i}}$ is the daily dose that needs to be evaluated, and N is the total number of samples.

The pharmacokinetic (PK) model developed by the University of California, San Francisco (i.e., Adult Vancomycin Dosing and Monitoring Recommendations), was used to benchmark our models. This PK model recommends different injection doses for patients under dialysis or not, and it has been published as an Excel toolkit online https://idmp.ucsf.edu/content/vancomycin-iv. The decision tree model developed by Imai et al. (2020) was implemented based on their publication.

### Model Availability

Our source codes and the models are publicly available on https://github.com/beverly0005/Vancomycin.

## Results

### Distribution of Vancomycin Trough Level

In the 3 years’ (Jan 2017 ∼ Dec 2019) data collected from Singapore General Hospital, we filtered out the records in which the duration between vancomycin lab test and its closest prior injection were not in the reasonable intervals (defined in Supplementary Appendix 1) because only the vancomycin lab tests at the specific time interval after injections was considered to properly reflect the impact of current injections, and considered as the vancomycin trough level. We used target trough vancomycin levels instead of the latest recommended AUC-guided approach. This was mainly because our institutional practice still adopts trough level–guided dosage titration, which is widely used in many institutions in the world. Hence, our dataset was only reflective of trough-based titration. There were 4,798 inpatient cases with 12,856 vancomycin injection records having vancomycin lab tests in the reasonable intervals. Based on the recommendations ^2^ (Rybak, 2006; Thomson et al., 2009) from several published studies and institutional practice, we set the desired therapeutic trough level of vancomycin to be in the range of 14 to 20 *mcg/ml.* Any deviation from that range may either reduce treatment efficacy or cause high risk of nephrotoxicity.

**FIGURE 3:**
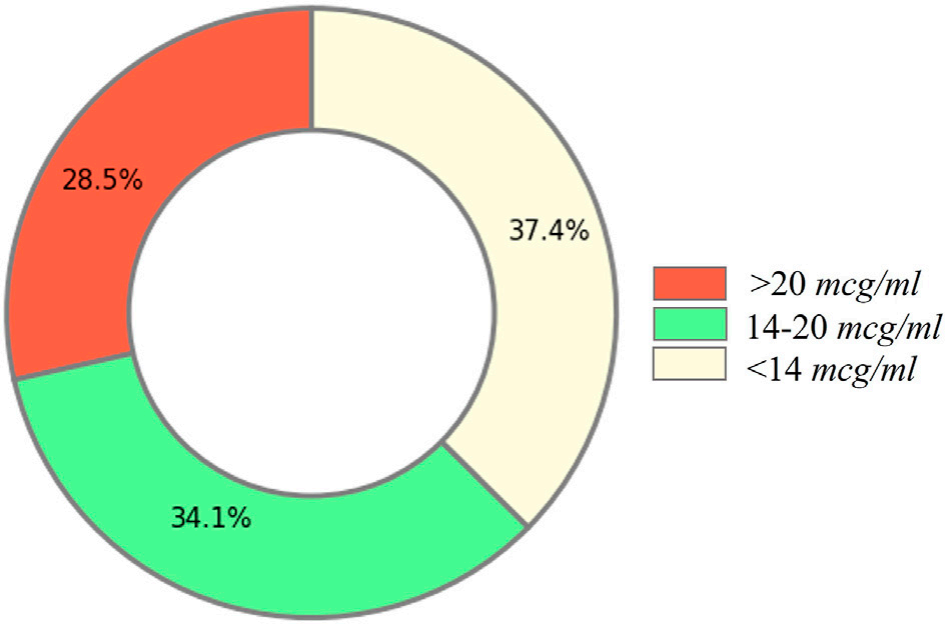
Distribution of the vancomycin trough level.

Distribution of the vancomycin trough level from 12,856 records is shown in Figure 3. Only a small portion (34.1%) of current injection doses can reach the desired vancomycin trough level, the rest injection doses were either too high or too low. This indicated that the current vancomycin injection practice needs to be improved in the hospital.

### Label and Cohort Generation

In order to train a supervised machine learning algorithm to recommend daily dose of vancomycin injection, we need to provide the label. In this study, we used a novel method to generate the derived label, the target daily dose for each record.

Theoretically the target daily dose should be the “appropriate” injection dose that leads to the desired therapeutic trough level of vancomycin for patients. However, the target daily dose could not be directly obtained from clinical data because it has high inter-individual variability and was unavailable for those whose vancomycin trough level fell outside the desired therapeutic range after injections. This is a common problem in developing dose recommender of vancomycin injection. Previous studies either used only the data of patients whose corresponding vancomycin trough levels fall into the desired therapeutic range (Huang et al., 2021), or set the “correct” injection dose proportionally to patients’ weight (Imai et al., 2020). These studies either neglected the potential to learn from failures, that is, injections with undesired therapeutic effect, or oversimplified inter-individual variability of injection dose.

We solved this issue by deriving the target daily dose for records based on the sequence of vancomycin injections and the corresponding vancomycin trough levels. The target daily dose is different for different patients, and even for the same patient, it may also be different if the injection is performed at different time. The detailed steps of deriving the target daily dose are described in Figure1 (see the *Method section*) and Supplementary Appendix 2 shows four examples of how target daily dose could be derived.

Since the target daily dose still could not be derived for some records using our method (case 3 in Supplementary Appendix 2) and might be invalid in some cases (case 4 in Supplementary Appendix 2), we generated the cohort by excluding those records. There were 56.0% patients and 61.5% records having a valid target daily dose. In our final cohort, there were 2,282 patients with 7,912 injection records. As mentioned in the *Method section*, the PAR (percentage of dose in the acceptable range) is a novel metric defined in our study to take into account whether the injection dose can drive the vancomycin trough level into the desired range. When measured using PAR, the PAR of the current injection practice was 67% in the final cohort, indicating that 67% injections were in the acceptable daily dose range. Specifically, PAR of the initial dose was 57% and PAR of the subsequent dose was 71%. Among those 67% injection records, as they were within the acceptable daily dose range of vancomycin injection, they were expected to achieve the desired vancomycin trough level if our definition of PAR metric was reasonable. Our analysis confirmed that 97.2% of them actually led to our desired vancomycin trough level in reality (Table2). This showed that our final cohort is clean and the derived label of each record was “appropriate.” It also indicated that our definition of PAR was consistent of the desired therapeutic vancomycin trough level. PAR makes more pharmacological sense compared to traditional metrics such as MAE or RMSE, hence we used it to evaluate the suggested dose.

**TABLE 2:** Actual vancomycin lab test result for records in an acceptable daily dose range (defined in the Method section).

Vancomycin test result	No. of records	%
(25, 28]	4	0.1
(20, 25]	30	0.6
[14, 20]	5,182	97.2
[10, 14)	104	2.0
[7.1, 10)	12	0.2

However, it should be noted that these PAR of the current injections in our final cohort were actually overestimated from reality. In reality without applying our cohort exclusion criteria, only 34.1% of vancomycin injection records could reach the desired therapeutic vancomycin trough level, as shown in Figure3. This is because the exclusion criteria of cohort formation removed the records without a target daily dose or an invalid target daily dose. For example, we had to exclude all the records from cases that failed to eventually reach the desired vancomycin trough level as no valid target daily dose could be derived for them.

### Descriptive Statistics

Key features of the cohort are summarized in Table3. The cohort was mostly composed of senior people, and 10% of them were undergoing hemodialysis when taking vancomycin injections. Regarding medicines taken by the patients, they were standardized into Anatomical Therapeutic Chemical Classification (ATC) of which the first level contains fourteen main anatomical and pharmacological groups. For example, the ATC code with the first level being “C” tells that the drug acts on cardiovascular system. In Table 3, the number of times medicines on the cardiovascular system dispensed means the number of dispensed records with ATC codes’ first level being “C.” Regarding latest lab tests, they refer to the latest lab tests done prior to vancomycin injections. These lab tests could be the ones immediately before the injections in the same visits, or could be the ones in much earlier visits. The missing rate of latest lab tests indicates that the patient has not performed those lab tests were not performed on patient in the past 1 year.

**TABLE 3:** Descriptive statistics of the study cohort.

Variable	% Missing	Mean (SD)	Interquantile [Q1, Q2]
Age (year)	0	63.1 (14.3)	[55, 73]
Female (Yes 1, No 0)	0	0.3 (0.5)	[0, 1]
Weight (kg)	0	64.7 (17.2)	[52.8, 73.0]
Hemodialysis (Yes 1, No 0)	0	0.1 (0.3)	[0, 0]
Number of times medicines on cardiovascular system dispensed in past 1 year	0	72.4 (114.5)	[7, 91]
Number of times medicines on alimentary tract or metabolism dispensed in past 1 year	0	144.1 (177.0)	[38, 180]
Number of times medicines on blood or blood forming organs dispensed in past 1 year	0	46.5 (76.0)	[6, 55]
Daily injection frequency	0	1.9 (0.5)	[2, 2]
Daily dose of vancomycin injection (*mg*)	0	2007.1 (989.8)	[1,250, 2,500]
Vancomycin trough level (*mcg/ml*)	0	17.1 (4.8)	[14.7, 19.3]
Latest serum creatinine (*micromoles/L*)	39.1	82.5 (78.3)	[45, 90]
Latest albumin (*g/L*)	39.0	28.7 (5.6)	[25, 32]
Latest eGFR	39.1	91.0 (33.2)	[71.2, 111.9]

### Model Development and Performance

**FIGURE 4:**
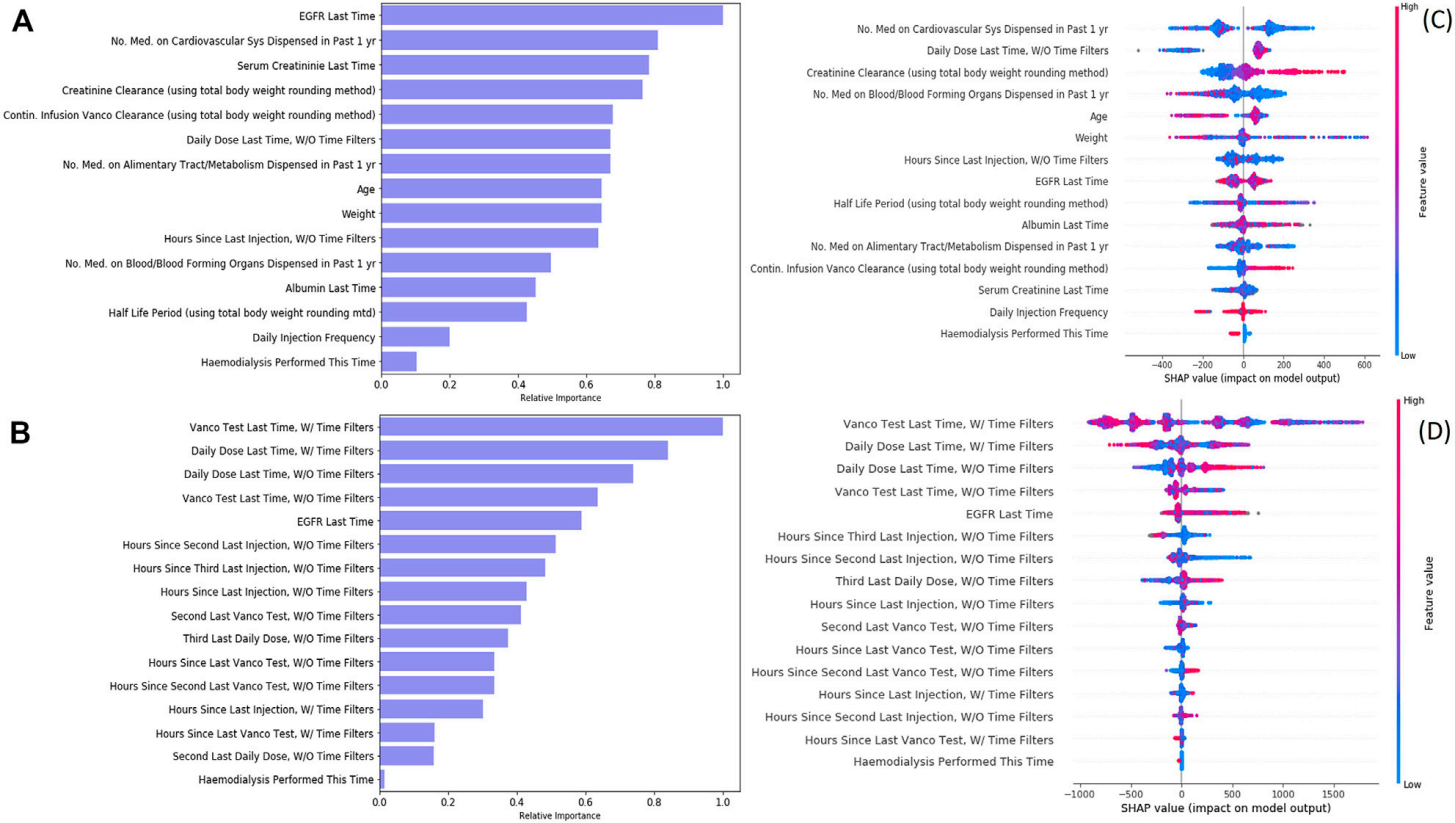
Feature importance and feature impact of vancomycin injection models. **(A)** Feature importance of the initial dose model. **(B)** Feature importance of the subsequent dose model. **(C)** SHAP result of the initial dose model. **(D)** SHAP result of the subsequent dose model.

To develop the machine learning models, we performed feature selection to select the most relevant features while maximizing the model performance (Fu and Lin, 2015). We started from 106 features, which included patients’ demographics, lab test history, diagnosis history, medication history, and the current clinical treatment. The feature selection process was done by repeatedly removing the features which are not important or not contributing to model performance, until the features in the final models are all important in prediction. Finally, we reduced to 15 features in the initial dose model and 16 features in the subsequent dose model (Figures 4A,B). Regarding the initial dose model, consistent with the PK model, weight, age, serum creatinine, creatinine clearance level, continuous infusion vancomycin clearance, and hemodialysis play important roles in prediction. Besides, our model shows that medication on the cardiovascular system, alimentary tract/metabolism, and blood forming organs also help in prediction.

During cohort generation, we applied filters to filter out the records to ensure we used a relatively clean cohort. However, data from excluded records may still be useful as derived features to improve the model performance. We therefore used two approaches to derive historical features (such as “vanco. test last time” and “daily dose last time”) used in the model training: one was features derived from the original dataset without applying the cohort exclusion criteria, the other was features derived from the filtered dataset after applying the cohort exclusion criteria, and the features derived were named accordingly by adding “w/time filter” (original dataset) and “w/o time filter” (filtered dataset). Our initial dose model had utilized, if any, the information on injection without filters in prediction. Our subsequent dose model used the history on both injections—with and without filters—and both vancomycin lab tests—with and without filters—in prediction. It showed that the historical sequence of injections and vancomycin lab tests could greatly improve the prediction power (PAR increased 21.7%).

A SHAP analysis was displayed (Figures 4C,D) to help interpret feature contributions to the daily dose prediction. Regarding initial dose model, creatinine clearance and continuous infusion vancomycin clearance are positively related to the predicted daily dose. On the contrast, the number of medication on blood or blood forming organs being dispensed and the procedure of hemodialysis are negatively related to the predicted daily dose. Regarding the subsequent dose model, although the latest injection (without time filter) seems to be positively related to the predicted daily dose, most of the features have a non-linear impact on the model recommendation.

Our model performance in the test set is shown in Table4. We compared the performance of our models with classical pharmacokinetic models and the machine learning model developed by Imai (only for initial dose) (Imai et al., 2020). Regarding the initial dose model, our model showed better PAR and MAE than the PK model and Imai’s model. As for the subsequent dose model, our model considered the historical injection and vancomycin lab test data and outperformed the PK model in both MAE and PAR. We also compared our model performance with the current practice in our cohort although the current practice performance in our cohort was largely overestimated than the reality in our hospital due to the filtering we had to apply to our study cohort. Our model still outperformed the current practice in subsequent injections as a higher percentage of injection doses suggested by our model were in the acceptable range. This shows the potential of our models to assist the decision on injection doses.

**TABLE 4:** Model performance of vancomycin injection (Test set).

Model	Metric	Current practice^a^	PK model	Imai 2020 model	Our model
Initial Dose	MAE (*mg/day*)	422.8	727.5	557.1	450.2
	PAR	56.0%	28.3%	43.8%	51.7%
Subsequent Dose	MAE (*mg/day*)	201.2	392.1		267.1
	PAR	72.8%	60.4%		73.4%

MAE: mean absolute error; PAR: percentage in the acceptable range.

a Please note that the performance of the current practice measured is overestimated (see *Result*, *Label and cohort generation*).

## Discussion

Optimizing vancomycin therapy remains a challenge in the current clinical practice owing to the dynamic profile of patients receiving the drug. Many factors are known to influence vancomycin dose–concentration response, including renal function, concomitant drugs, and weight. In clinical practice, various approaches have been used to guide clinicians in vancomycin dosing such as dosing nomograms and Bayesian estimation methods. In a study by Huang et al., a vancomycin dose prediction model was established using eXtreme Gradient Boosting (XGBoost) for feature selection and model construction (Huang et al., 2021). Their model did not differentiate between initial dose and subsequent dose predictions. The study selected variables that were similar to those in our study, namely renal function, weight, and concomitant drugs. This finding is also in line with published pharmacokinetic studies (Monteiro et al., 2018; Kim et al., 2019; Lin et al., 2021), whereby eGFR or estimated creatinine clearance and weight are key predictors of vancomycin dosage requirements. The overall model performance in Huang et al.’s study was reported to be 70.2%.

In this study, we developed and validated a promising approach using machine learning to guide dosing decisions. Based on our test results, our machine learning based models performed better than the PK model we tested in vancomycin dose recommendation. The PAR of our initial dose model was 51.7%, and our subsequent dose model was 73.4% in our retrospective cohort. Since all patients in our test data set were not seen by the models during the training process, we expect that the performance of our machine learning models can generalize to new patients who will be admitted in this hospital in the future (a prospective study is needed to test our hypothesis).

**FIGURE 5:**
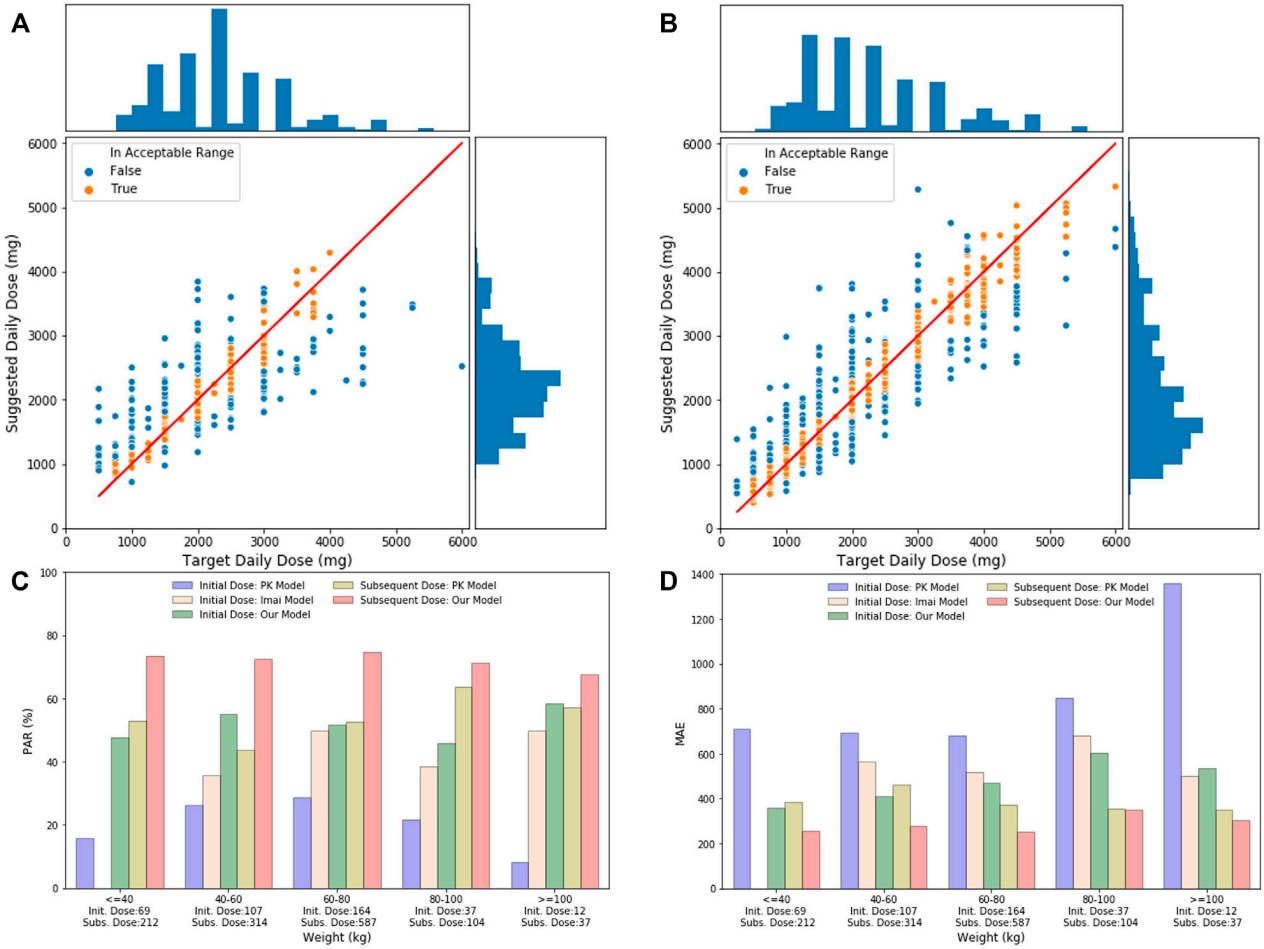
**(A)** Initial daily dose suggested by our models and the target daily dose in the test set. The solid line indicates perfect match where the suggested daily dose equals to the target daily dose. The bar chart on the top is the distribution of target daily dose, and the bar chart on the right shows the distribution of suggested daily dose. **(B)** Subsequent daily dose suggested by our models and the target daily dose in the test set. **(C)** Comparison of PAR of different models, including the PK initial dose model, Imai model, our initial model, PK subsequent model, and our subsequent model, stratified by patient’s weight groups in test data. Number of records in each weight group is shown under the *x*-axis. **(D)** Comparison of MAE of different models, including the PK initial dose model, Imai model, our initial model, PK subsequent model, and our subsequent model, stratified by patient’s weight groups in test data.

As indicated in Figures 5A,B, the distribution of suggested daily doses and target daily doses was similar to each other for both the initial dose and the subsequent dose. Based on these scatter plots, our initial dose model does not perform well when target daily dose is in extreme value (i.e., <750 mg/day or >4000 mg/day). However, when stratified the test data by weight, the PAR and MAE of our models were stable across different weight groups. The performance of our models was better than the PK model and Imai’s model across all the weight groups, while Imai’s model can only apply to the initial dose with weight ≥ 40 kg.

In order to understand where our model did not perform well, we analyzed some examples which the suggested daily dose by our models had relatively larger differences from the target daily dose. In the initial dose examples (Table 5), the suggested dose by our model was higher than the target daily dose mainly due to the heavy weight of patients. The smaller injection dose in the current practice was likely to be given based on the expertise of doctors or based on some unseen data which was not accessible by a current study. In the subsequent dose examples (Table 6), the suggested dose by our model, although different from the target titration pathway derived from the current practice, still seemed to be a reasonable titration pathway to achieve the therapeutic effect, as reviewed by an experienced pharmacist. This indicates an opportunity for future research in this aspect.

**TABLE 5:** Examples of the initial dose model showing different target daily doses and suggested daily doses.

case No	Time	Age (yr)	Weight (kg)	Vanco. trough level (*mcg/ml*)	Daily dose (*mg/day*)		
					Current practice	Target	Suggestion by model
6718324219D^1^	April 1, 2018 15:28	41	90.4	17.6	2000	2000	3,553.8
6719396480E^1^	December 10, 2019 17:18	60	113	14.2	2000	2000	3,840.9

Note: Patients in these two cases are not under the treatment of hemodialysis.

**TABLE 6:** Examples of the subsequent dose model showing different target daily doses and suggested daily doses.

case No	Time	Vanco. last time		Hours since last injection		Vanco. trough level (*mcg/ml*)	Daily dose (*mg/day*)		
		Daily dose	Trough level	Last injection	Vanco. test		Current practice	Target	Suggestion by model
6719300968D	January 7, 2019 16:20	2000	5.9	38.5	27.0	7.3	2,500	4,500	3,615.9
6719300968D	January 9, 2019 5:30	2,500	7.3	37.2	25.3	14.1	4,500	4,500	4,083.5
6719300968D	January 10, 2019 13:08	4,500	14.1	31.6	25.4	18.5	3,500	3,500	4,760.1
6719300968D	January 12, 2019 20:58	3,500	18.5	55.8	46.5	18.0	5,250	5,250	3,892.5

Taken collectively, machine learning can potentially augment clinician decision making better than existing pharmacokinetic models. This is especially useful to guide decision-making for inexperienced doctors or pharmacists in making consistent and safe dosing recommendations for high-risk medications like vancomycin.

## Data Availability

The original contributions presented in the study are included in the article/Supplementary Material; further inquiries can be directed to the corresponding author.

## Footnotes

1 More information on eHints can be found on the website: https://www.ihis.com.sg/Project_Showcase/Healthcare_Systems/Pages/eHINTS.aspx.

2 British National Formulary (BNF) recommends 10–15 *mcg/ml* for therapeutic vancomycin trough level and 15–20 *mcg/ml* for more resistant strains. The American Thoracic Society published guidelines to recommend vancomycin trough level of 15–20 *mcg/ml* to treat methicillin-resistant *S. aureus* pneumonia. In the study, we set the desired therapeutic vancomycin trough level to 14–20 *mcg/ml* with more emphasis on treatment of complicated infection.
