# Supplementary material for: AI Models to Assist Vancomycin Dosage Titration
Source: Front Pharmacol. 2022 Feb 8;13:801928. doi: 10.3389/fphar.2022.801928 (PMC8861296; doi:10.3389/fphar.2022.801928)
Supplement: Supplementary file 1 [file DataSheet2.docx]

**Appendix 1. Definition on Corresponding Vancomycin Lab Test to Injection**

It takes time for vancomycin injections to take effect. Based on pharmacokinetic principles, it takes up to 5 half-lives (one half-life of vancomycin = 4-6 hours) before steady state concentration is achieved. Vancomycin trough level measured at the specified time interval in Table A1 after injections is considered to properly reflect the impact of injections.

**Table A1. Definition on corresponding vancomycin lab test**

| Daily Injection Frequency | Vancomycin Lab Test Done in Hours after Last Injection Performed |
| --- | --- |
| 1 | [20, 28] |
| 2 | [9, 15] |
| 3 | [6, 10] |
| 4 | [5, 7] |

***Notes***: If one injection has multiple vancomycin lab tests done in the specified time interval, the lab test with the lowest vancomycin trough level will be used as the corresponding vancomycin lab test.

Daily injection frequency is not available in the data. We derive it as the number of injections done in the past 20 hours (including this time). However, if the earliest injection is done less than 20 hours before, then we will use the number of injections done in the next 20 hours as daily injection frequency. In our cohort, daily injection frequency ranges from 1 to 4, with most patients being 2.

**Appendix 2. Examples of Target Daily Dose**

Table A2 shows some examples of how target daily dose can be defined. For Case No. 1, the target daily dose equals to its current daily dose because the vancomycin trough level is in the desired therapeutic range 14-20 *mcg/ml*. For Case No. 2, the vancomycin trough levels of the injection at both 11/11/2019 19:58 and 12/11/2019 8:15 are outside the therapeutic range. The target daily doses of these two days therefore take the value from current daily dose (750 mg) of the injection done at 14/11/2019 10:06. For Case No. 3, the target daily dose cannot be defined because there is no injection afterwards with vancomycin trough level in desired therapeutic range. For Case No. 4, target daily dose at 29/3/2017 19:53 is invalid because it is smaller than current daily dose with vancomycin trough level < 14 mcg/ml (please refer to condition *vi* in cohort exclusion in Methods).

**Table A2. Examples of target daily dose**

| **Case No.** | **Injection** | | **Corresponding Vanco. Lab Test** | | **Target Daily Dose** |
| --- | --- | --- | --- | --- | --- |
|  | **Timestamp** | **Current Daily Dose** | **Timestamp** | **Trough Level** |  |
| 1 | 13/1/2017 19:32 | 2000 *mg* | 14/1/2017 5:00 | 16.9 *mcg/ml* | 2000 *mg* |
| 2 | 11/11/2019 19:58 | 1000 *mg* | 12/11/2019 8:58 | 21.6 *mcg/ml* | 750 *mg* |
| 2 | 12/11/2019 8:15 | 1000 *mg* | 13/11/2019 3:13 | 20.7 *mcg/ml* | 750 *mg* |
| 2 | 14/11/2019 10:06 | 750 *mg* | 15/11/2019 9:47 | 15.6 *mcg/ml* | 750 *mg* |
| 2 | 16/11/2019 9:00 | 1000 *mg* | 16/11/2019 20:17 | 16.1 *mcg/ml* | 1000 *mg* |
| 3 | 6/1/2018 19:10 | 1250 *mg* | 6/1/2018 23:50 | 12.1 *mcg/ml* | -- |
| 3 | 8/1/2018 10:21 | 1500 *mg* | 8/1/2018 16:33 | 13.5 *mcg/ml* | -- |
| 4 | 29/3/2017 19:53 | 2000 *mg* | 30/3/2017 6:37 | 5.0 *mcg/ml* | 1500 *mg (Invalid)* |
| 4 | 2/4/2017 19:13 | 1500 *mg* | 3/4/2017 4:26 | 16.7 *mcg/ml* | 1500 *mg* |
| 4 | 9/4/2017 8:11 | 1000 *mg* | 10/4/2017 4:50 | 16.9 *mcg/ml* | 1000 *mg* |
