# Supplementary material for: AI Models to Assist Vancomycin Dosage Titration
Source: Front Pharmacol. 2022 Feb 8;13:801928. doi: 10.3389/fphar.2022.801928 (PMC8861296; doi:10.3389/fphar.2022.801928)
Supplement: Supplementary file 2 [file DataSheet1.docx]

**Table S1. List of All Features**

| **S/N** | **Feature** | **Type** |
| --- | --- | --- |
| 1 | Race_Chinese | Boolean |
| 2 | Race_Malay | Boolean |
| 3 | Race_Indian | Boolean |
| 4 | Race_Others | Boolean |
| 5 | Female | Boolean |
| 6 | Age | Float |
| 7 | Height | Float |
| 8 | Weight | Float |
| 9 | Last Vancomycin Trough Level, With Time Filter | Float |
| 10 | Duration Since Last Vancomycin Lab Test, With Time Filter | Float |
| 11 | Last Vancomycin Trough Level, Without Time Filter | Float |
| 12 | Duration Since Last Vancomycin Lab Test, Without Time Filter | Float |
| 13 | Second Last Vancomycin Trough Level, With Time Filter | Float |
| 14 | Duration Since Second Last Vancomycin Lab Test, With Time Filter | Float |
| 15 | Second Last Vancomycin Trough Level, Without Time Filter | Float |
| 16 | Duration Since Second Last Vancomycin Lab Test, Without Time Filter | Float |
| 17 | Injection Frequency | Integer |
| 18 | Last Daily Dose of Vancomycin Injection, With Time Filter | Float |
| 19 | Duration Since Last Vancomycin Injection, With Time Filter | Float |
| 20 | Last Daily Dose of Vancomycin Injection, Without Time Filter | Float |
| 21 | Duration Since Last Vancomycin Injection, Without Time Filter | Float |
| 22 | Second Last Daily Dose of Vancomycin Injection, With Time Filter | Float |
| 23 | Duration Since Second Last Vancomycin Injection, With Time Filter | Float |
| 24 | Second Last Daily Dose of Vancomycin Injection, Without Time Filter | Float |
| 25 | Duration Since Second Last Vancomycin Injection, Without Time Filter | Float |
| 26 | Third Last Daily Dose of Vancomycin Injection, With Time Filter | Float |
| 27 | Duration Since Third Last Vancomycin Injection, With Time Filter | Float |
| 28 | Third Last Daily Dose of Vancomycin Injection, Without Time Filter | Float |
| 29 | Duration Since Third Last Vancomycin Injection, Without Time Filter | Float |
| 30 | Duration Btw Last Vancomycin Injection and Last Vancomycin Lab Test, With Time Filter | Float |
| 31 | Duration Btw Second Last Vancomycin Injection and Second Last Vancomycin Lab Test, With Time Filter | Float |
| 32 | Last Serum Creatinine Level | Float |
| 33 | Second Last Serum Creatinine Level | Float |
| 34 | Last Bilirubin Level | Float |
| 35 | Last Alkaline Phosphatase Level | Float |
| 36 | Last Alanine Aminotransferase Level | Float |
| 37 | Last Aspartate Aminotransferase Level | Float |
| 38 | Last Albumin Level | Float |
| 39 | Last White Blood Cell Level | Float |
| 40 | Last C-Reactive Protein Level | Float |
| 41 | Last Procalcitonin Level | Float |
| 42 | Last Culture Test Level | Float |
| 43 | Last eGFR Level | Float |
| 44 | Second Last eGFR Level | Float |
| 45 | Haemodialysis | Boolean |
| 46 | Peritoneal | Boolean |
| 47 | No. Diseases in Charlson Comorbidity Index (CCI) Being Diagnosed Before | Integer |
| 48 | No. Times Being Diagnosed With Severe Liver Diseases (CCI) | Integer |
| 49 | No. Times Being Diagnosed With Diabetes-Long Term Complication (CCI) | Integer |
| 50 | No. Times Being Diagnosed With Renal Diseases (CCI) | Integer |
| 51 | No. Times Being Diagnosed With Pulmonary Diseases (CCI) | Integer |
| 52 | No. Times Being Diagnosed With Peptic Ulcer (CCI) | Integer |
| 53 | No. Times Being Diagnosed With Cerebral Vascular Accident (CCI) | Integer |
| 54 | No. Times Being Diagnosed With Myocardial Infarction (CCI) | Integer |
| 55 | No. Times Being Diagnosed With Congestive Heart Failure (CCI) | Integer |
| 56 | No. Times Being Diagnosed With Metastatic Cancer (CCI) | Integer |
| 57 | No. Times Being Diagnosed With Connective Tissue Disorder (CCI) | Integer |
| 58 | No. Times Being Diagnosed With Mild/Moderate Liver Diseases (CCI) | Integer |
| 59 | No. Times Being Diagnosed With Hemiplegia/Paraplegia (CCI) | Integer |
| 60 | No. Times Being Diagnosed With Cancer (CCI) | Integer |
| 61 | No. Diseases in Chronic Disease Mgmt. System (CDMS) Being Diagnosed Before | Integer |
| 62 | No. Times Being Diagnosed With COPD (CDMS) | Integer |
| 63 | No. Times Being Diagnosed With Schizophrenia (CDMS) | Integer |
| 64 | No. Times Being Diagnosed With Dementia (CDMS) | Integer |
| 65 | No. Times Being Diagnosed With Suspected Stroke (CDMS) | Integer |
| 66 | No. Times Being Diagnosed With Coronary Heart Disease (CDMS) | Integer |
| 67 | No. Times Being Diagnosed With Diabetes Mellitus (CDMS) | Integer |
| 68 | No. Times Being Diagnosed With Asthma (CDMS) | Integer |
| 69 | No. Times Being Diagnosed With Heart Failure (CDMS) | Integer |
| 70 | No. Times Being Diagnosed With Hypertension (CDMS) | Integer |
| 71 | No. Times Being Diagnosed With Renal Disease (CDMS) | Integer |
| 72 | No. Times Being Diagnosed With Hip Fracture (CDMS) | Integer |
| 73 | No. Times Being Diagnosed With Abnormal Weight (CDMS) | Integer |
| 74 | No. Times Being Diagnosed With Osteoporosis (CDMS) | Integer |
| 75 | No. Times Being Diagnosed With General Anxiety Disorder (CDMS) | Integer |
| 76 | No. Times Being Diagnosed With Stroke (CDMS) | Integer |
| 77 | No. Times Being Diagnosed With Spine Fracture (CDMS) | Integer |
| 78 | No. Times Being Diagnosed With Dyslipidaemia (CDMS) | Integer |
| 79 | No. Times Medicines on Dermatology Being Dispensed in Past 1 Year | Integer |
| 80 | No. Times Medicines on Nervous System Being Dispensed in Past 1 Year | Integer |
| 81 | No. Times Medicines on Blood Being Dispensed in Past 1 Year | Integer |
| 82 | No. Times Medicines on Alimentary Tract/Metabolism Being Dispensed in Past 1 Year | Integer |
| 83 | No. Times Medicines on Cardiovascular System Being Dispensed in Past 1 Year | Integer |
| 84 | No. Times Medicines on Anti-infective Being Dispensed in Past 1 Year | Integer |
| 85 | No. Times Medicines on Respiratory System Being Dispensed in Past 1 Year | Integer |
| 86 | No. Times Medicines on Other Diseases Being Dispensed in Past 1 Year | Integer |
| 87 | No. Times Medicines on Muscular-skeletal System Being Dispensed in Past 1 Year | Integer |
| 88 | No. Times Medicines on Diabetes Being Dispensed in Past 1 Year | Integer |
| 89 | No. Times Medicines on Sensory Organs Being Dispensed in Past 1 Year | Integer |
| 90 | No. Times Medicines on Genital-urinary/Sex Hormones Being Dispensed in Past 1 Year | Integer |
| 91 | No. Times Medicines on L: Antineoplastic and Immunomodulation Being Dispensed in Past 1 Year | Integer |
| 92 | No. Times Medicines on Systemic hormonal preparations Being Dispensed in Past 1 Year | Integer |
| 93 | Elderly Flag (PK Model) | Boolean |
| 94 | Vd_vanc Parameter (PK Model) | Float |
| 95 | Creatinine Clearance using Total Body Weight (PK Model) | Float |
| 96 | Creatinine Clearance using Adjusted Body Weight (PK Model) | Float |
| 97 | Creatinine Clearance using Ideal Body Weight (PK Model) | Float |
| 98 | Creatinine Clearance using Total Body Weight Rounding Method (PK Model) | Float |
| 99 | Clvanc Parameter using Total Body Weight (PK Model) | Float |
| 100 | Clvanc Parameter using Adjusted Body Weight (PK Model) | Float |
| 101 | Clvanc Parameter using Ideal Body Weight (PK Model) | Float |
| 102 | Clvanc Parameter using Total Body Weight Rounding Method (PK Model) | Float |
| 103 | Half Life Period using Total Body Weight (PK Model) | Float |
| 104 | Half Life Period using Adjusted Body Weight (PK Model) | Float |
| 105 | Half Life Period using Ideal Body Weight (PK Model) | Float |
| 106 | Half Life Period using Ideal Body Weight Rounding Method (PK Model) | Float |

**Table S2. Model Comparison**

| **Model** | **Data** | **Metric** | **LightGBM** | **XGBoost** | **SVR** | **LR** |
| --- | --- | --- | --- | --- | --- | --- |
| Initial Dose | Validation | MAE (mg/day) | 442.6 | 452.8 | 579 | 24332.9 |
|  |  | PAR | 51.3% | 50.3% | 34.6% | 0.6% |
| Subsequent Dose | Validation | MAE (mg/day) | 287.6 | 304.1 | 354.1 | 332.7 |
|  |  | PAR | 72.7% | 71.8% | 63.1% | 63.6% |

Notes: MAE is mean absolute error, PAR is the percentage in the acceptable range, SVR is supporting vector regression, and LR is linear regression.
